# Gene silencing via DNA methylation in naturally occurring *Tragopogon miscellus* (Asteraceae) allopolyploids

**DOI:** 10.1186/1471-2164-15-701

**Published:** 2014-08-22

**Authors:** Tina Sehrish, V Vaughan Symonds, Douglas E Soltis, Pamela S Soltis, Jennifer A Tate

**Affiliations:** Institute of Fundamental Sciences, Massey University, Palmerston North, New Zealand; Department of Biology, University of Florida, Gainesville, Florida USA; Florida Museum of Natural History, University of Florida, Gainesville, Florida USA

**Keywords:** Allopolyploidy, DNA methylation, Gene silencing, *Tragopogon*, Whole-genome duplication

## Abstract

**Background:**

Hybridization coupled with whole-genome duplication (allopolyploidy) leads to a variety of genetic and epigenetic modifications in the resultant merged genomes. In particular, gene loss and gene silencing are commonly observed post-polyploidization. Here, we investigated DNA methylation as a potential mechanism for gene silencing in *Tragopogon miscellus* (Asteraceae), a recent and recurrently formed allopolyploid. This species, which also exhibits extensive gene loss, was formed from the diploids *T. dubius* and *T. pratensis*.

**Results:**

Comparative bisulfite sequencing revealed CG methylation of parental homeologs for three loci (S2, S18 and TDF-44) that were previously identified as silenced in *T. miscellus* individuals relative to the diploid progenitors. One other locus (S3) examined did not show methylation, indicating that other transcriptional and post-transcriptional mechanisms are likely responsible for silencing that homeologous locus.

**Conclusions:**

These results indicate that *Tragopogon miscellus* allopolyploids employ diverse mechanisms, including DNA methylation, to respond to the potential shock of genome merger and doubling.

**Electronic supplementary material:**

The online version of this article (doi:10.1186/1471-2164-15-701) contains supplementary material, which is available to authorized users.

## Background

Whole-genome duplication (polyploidy) has played a major role in eukaryotic evolution [1–6]. In particular, flowering plants have experienced repeated episodes of polyploidy since they shared a common ancestor with the gymnosperms some 300 million years ago [7, 8]. Understanding the genomic consequences of polyploidization, particularly when accompanied by hybridization (allopolyploidy), allows insight into the potential for speciation and adaptation of these novel entities [9, 10]. In particular, the merger and doubling of two divergent genomes can induce different genetic and epigenetic changes in the resulting polyploid [11–16]. Genetic modifications can include gene loss, genome down-sizing, variable mutation rates of the duplicated genes (homeologs), chromosomal rearrangements and regulatory incompatibilities resulting from post-transcriptional modifications in the merged genomes [16–24]. Epigenetic modifications involve heritable changes in gene expression without changes in the nucleotide sequence [25–27] and can include histone modification, DNA methylation, chromatin remodeling, or microRNA or prion activity [28–30]. DNA methylation, the addition of a methyl group at position 5 of the pyrimidine ring of cytosine, is a common mechanism associated with gene silencing in polyploids [31–33]. In general, cytosine methylation is important for maintaining genomic stability and is involved in genomic imprinting, transposon silencing and epigenetic regulation of gene transcription [30, 34–36].

Here, we investigated gene silencing via methylation in the allotetraploid plant *Tragopogon miscellus*. This species formed repeatedly during the early 1900s in the western United States, following the introduction of the diploid progenitors, *T. dubius* and *T. pratensis*, from Europe [37–40]. Previous studies identified extensive homeolog loss [21, 41–43] and chromosomal variation [17] in naturally occurring *T. miscellus* populations. Two studies [42, 43] also identified homeologous gene silencing in some individuals of *T. miscellus*, but the mechanism for silencing was not known. In Tate et al. [43], the *T. dubius* copy of one locus (TDF-44) was silenced in multiple individuals from Pullman, Washington, and Moscow, Idaho. In Buggs et al. [42], six loci showed variable silencing of *T. dubius* or *T. pratensis* homeologs in a few individuals from five different populations (Oakesdale, Pullman, and Spangle, Washington; Moscow and Garfield, Idaho). In the present study, we used comparative bisulfite sequencing to determine if these loci were silenced by methylation.

## Results and discussion

### CG methylation regulates duplicate gene expression

**Figure 1:**
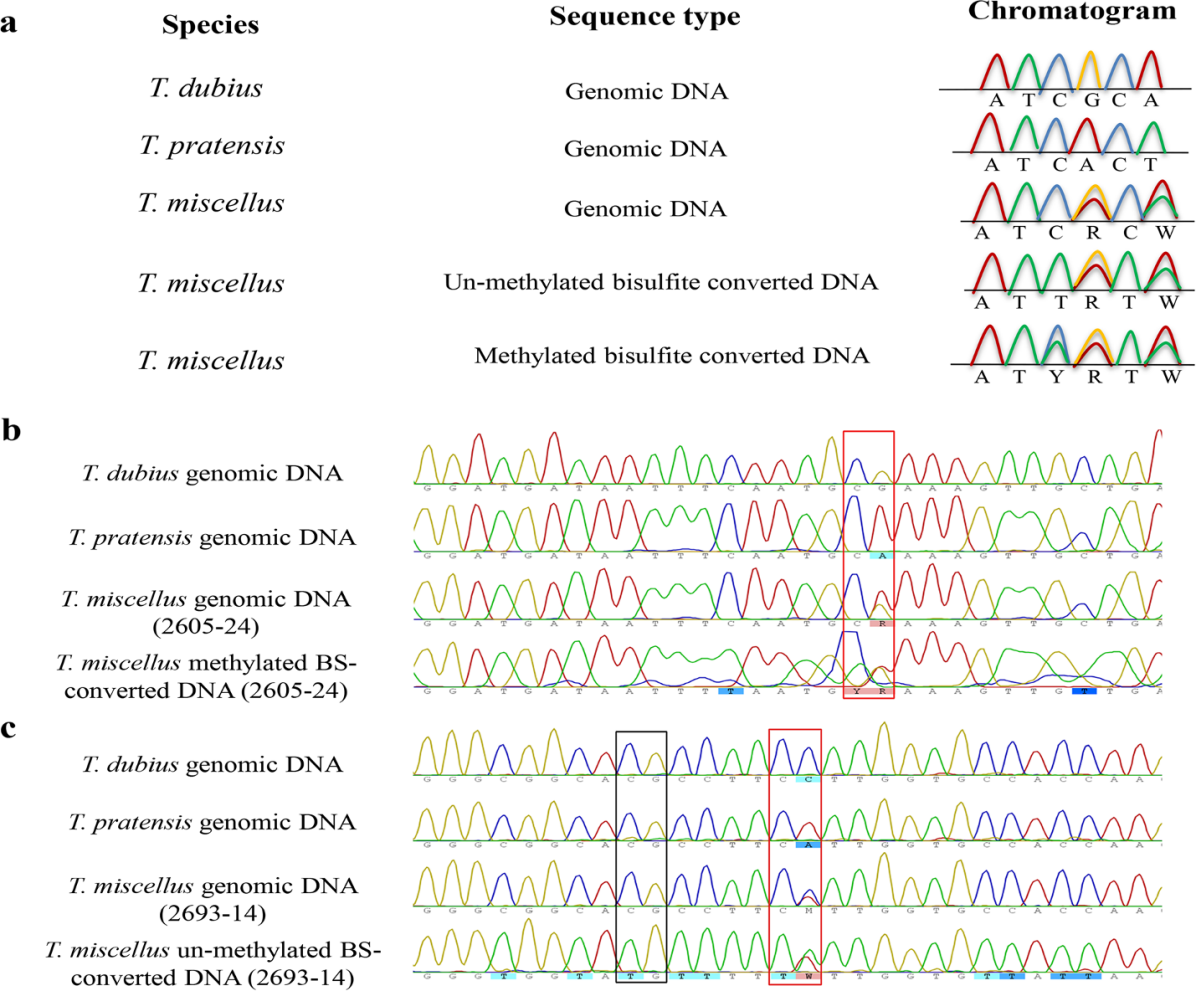
**Methylation of homeologous loci in** ***Tragopogon miscellus.*** Sequence polymorphisms between the diploid parents (*Tragopogon dubius* and *T. pratensis*) were used to determine homeolog-specific silencing in *T. miscellus* allopolyploids. **(a)** Diagrammatic illustration of the expected chromatogram peaks for genomic and bisulfite-converted sequences when un-methylated or methylated in allopolyploid *T. miscellus*. This example shows silencing of the *T. dubius* homeolog. **(b)** Chromatograms of TDF-44 indicating the position of a methylated CG adjacent to a polymorphic site (red box) in *T. miscellus* compared to the diploids. **(c)** Chromatograms of S18 showing an un-methylated CG site in *T. miscellus* (black box) and the location of a polymorphic site between parental copies (red box). Red, blue, green and yellow colors of the chromatogram correspond to A, C, T and G, respectively. IUPAC ambiguity codes: W = A/T, Y = C/T, R = A/G. BS-converted = bisulfite-converted.

Genomic and bisulfite-converted sequences were acquired for four loci [TDF-44 (putative leucine-rich repeat transmembrane protein kinase) [43], S2 (putative RNA binding protein), S3 (putative NADP/FAD oxidoreductase), and S18 (putative porphyrin-oxidoreductase) [42]] from allopolyploid *Tragopogon miscellus* and the diploid parents *T. dubius* and *T. pratensis* (Table 1). A fifth locus (S8, putative acetyl transferase) identified as silenced in Buggs et al. [42] was not amenable for study because no single nucleotide polymorphisms (SNPs) between the diploids were maintained following bisulfite conversion to distinguish the parental copies in the allopolyploid (Figure 1a). For the four loci examined, we took advantage of SNPs between the diploids to determine if a parental homeolog was silenced by methylation in the *T. miscellus* individuals. In addition to the partial gene sequences retrieved in the two previous studies [42, 43], 5’ genome walking was undertaken to determine methylation status of the promoter regions. The new sequences were deposited in GenBank (KM260156-KM260165).

**Table 1:** **Results of methylation analysis**

Population	Species	Lineage	Locus silenced	Methylated? ^a^
Pullman	*T. dubius*	2613-1	NA	
	*T. dubius*	2613-11	NA	
	*T. miscellus*	2605-4	TDF44_d_	Yes-A/S
	*T. miscellus*	2605-7	TDF44_d_	Yes-A/S
	*T. miscellus*	2605-13	TDF44_d_	Yes-A/S
			S18_d_	Yes-A
	*T. miscellus*	2605-24	TDF44_d_	Yes-A/S
	*T. miscellus*	2605-28	TDF44_d_	Yes-A/S
	*T. miscellus*	2605-46	TDF44_d_	Yes-A/S
Moscow	*T. pratensis*	2608-31	NA	
	*T. pratensis*	2608-35x	NA	
	*T. miscellus*	2604-4	TDF44_d_	Yes-A/S
	*T. miscellus*	2604-11	TDF44_d_	Yes-A/S
	*T. miscellus*	2604-15	TDF44_d_	Yes-A/S
	*T. miscellus*	2604-22	*T. pratensis genomic copy lost	
	*T. miscellus*	2604-24	TDF44_d_	Yes-A/S
	*T. miscellus*	2604-35	TDF44_d_	Yes-A/S
Spangle	*T. miscellus*	2693-7	S3_p_	No
	*T. miscellus*	2693-8	S3_p_	No
	*T. miscellus*	2693-14	S3_p_	No
			S18_d_	No
Garfield	*T. dubius*	2687-11	NA	
	*T. pratensis*	2689-17	NA	
	*T. miscellus*	2688-3	S2_d_	Yes-A/S
			S3_d_	No
			S18_d_	No
Oakesdale	*T. miscellus*	2671-11	S2_d_	Yes-A/S

NA = Not applicable; subscript d or p indicates the homeolog silenced; ^a^A = antisense strand, S = sense strand.

Individual plants used in the study and their methylation status for the genes studied; silencing data from [42, 43].

Inspection of the promoter and coding regions identified CG sites, which are common methylation sites in plants [44, 45]. The integrity of bisulfite conversion was determined from the conversion of all the Cs not adjacent to a G into Ts. The loci studied here all showed complete bisulfite conversion in the genic regions, while incomplete conversion at a few sites was detected in the promoter region of TDF-44 for three polyploid individuals (2604-4, 2604-35 and 2605-14). Given that most of the promoter and genic regions were properly converted, the incomplete conversion for TDF-44 does not influence the overall interpretation of the results. Such low frequency of partial bisulfite conversion is commonly due to reaction temperature [46, 47]. Alternatively, these sites could represent varying levels of CHH (H = A, C, or T) or CHG methylation [48].

CG methylation of both sense and antisense strands was detected in the genic and promoter regions of S2 (putative RNA binding protein) and TDF-44 (putative leucine-rich repeat transmembrane protein kinase). TDF-44 included seven CG sites in the promoter region and four in genic regions; the *T. dubius* homeolog was methylated in 11 of 12 *T. miscellus* individuals from Pullman and Moscow (Figure 1b, Table 1), which reveals the mechanism of silencing observed in Tate et al. [43]. The exception was individual 2604-22, which retained only the *T. dubius* genomic homeolog and therefore expressed that copy [43]. Similarly, we found methylation of the S2 locus, which was shown by Buggs et al. [42] to be silenced in two *T. miscellus* individuals (one each from Garfield and Oakesdale). All CG sites in the promoter (four) and genic (six) regions of S2 were methylated in both individuals. However, both parental homeologs showed CG methylation and sequencing the cloned bisulfite-converted sequences revealed twice as many methylated *T. dubius* cloned copies as *T. pratensis* copies. This result suggests that methylation can quantitatively regulate the level of expression of parental copies rather than completely silencing one homeolog. For locus S18 (putative porphyrin-oxidoreductase), which had 11 CG sites in the promoter and 11 in the genic regions, the only methylation detected was one individual that showed hemimethylation of the antisense strand (Table 1). Interestingly, this individual only showed methylation at five of the 11 CG sites in the genic regions.

Analysis of the promoter and genic regions of the other locus (S3-putative NADP/FAD oxidoreductase) did not show methylation of any of the CG sites (Figure 1c, Table 1; S3 included six CG sites in the promoter and three CG sites in the genic regions). Thus, there may be mechanisms other than DNA methylation that are responsible for homeolog-specific silencing. For example, histone deacetylation (causing chromatin condensation) is thought to be responsible for transcriptional repression [49–51]. RNA interference (RNAi) is also widely associated with post-transcriptional silencing via a number of different mechanisms, including mRNA degradation, translational inhibition and the repression of transcription elongation [52–55].

Natural variation in epigenetic patterning is not well understood, but can be an important driver of ecological speciation, as has been found in *Viola* [56] and *Dactylorhiza* [57, 58]. Here we find differences in the methylation status and silencing mechanisms in allopolyploid individuals from different populations (Table 1). For the 17 *Tragopogon miscellus* polyploids studied here, most showed silencing of only one locus in the previous studies of Tate et al. [43] and Buggs et al. [42], but three individuals showed silencing of two or more loci. Some of these loci are silenced by methylation, but others are not, suggesting diverse mechanisms exist within an allopolyploid individual to regulate duplicate gene expression. For the loci that were methylated, two showed 100% CG methylation in genic and promoter regions (TDF-44 and S2), while the third was methylated at 50% of the genic CG sites. As methylation of gene regions is not usually associated with gene silencing in plants [48], how this pattern of methylation contributes to silencing this gene, if at all, is not understood. Comparison of the methylation status of silenced vs. unsilenced loci could lend further insight into the role of gene body methylation in *Tragopogon*.

Hence, as in other polyploid species [*Spartina anglica*, [11], *Brassica*, [59], wheat, [60], rice, [61], *Arabidopsis suecica*, [62]], genome evolution in *Tragopogon miscellus* includes DNA methylation as a mechanism to regulate duplicate gene expression, which we demonstrate here for the first time. Previous studies in *Tragopogon* showed homeolog loss [41–43] and chromosomal repatterning [63, 64] following allopolyploid formation. These latter phenomena seem to be more common mechanisms in *T. miscellus* populations than expression changes for dealing with the ‘genome shock’ that accompanies hybridization and whole genome duplication [65]. The loci silenced via methylation had the *T. dubius* copy silenced, which, although a small number, may indicate a ‘preference’ for silencing loci of one progenitor’s genome. This result is true of the *T. miscellus* polyploids formed with either *T. dubius* (Pullman) or *T. pratensis* (Garfield, Moscow, Oakesdale, Spangle) as the maternal parent, so there does not seem to be a maternal ‘imprinting’ influence for the loci studied here. This interpretation is in line with previous studies that have reported a greater tendency of homeolog loss of the *T. dubius* copy compared to *T. pratensis* [21, 41–43, 66–68]. Curiously, in the case of rDNA, although *T. dubius* homeologs are more frequently lost from the polyploid genomes, transcription rates of remaining *T. dubius* copies are higher than *T. pratensis* copies [67]. As *T. miscellus* has shown a high frequency of homeolog loss, but little gene silencing based on the studies to date [21, 41–43, 69], a more comprehensive genome-wide analysis of methylation would help to determine the role of this epigenetic mechanism in shaping the evolution of *Tragopogon* allopolyploid genomes.

## Conclusions

Allopolyploids can employ diverse mechanisms to cope with duplicate and redundant genomes. While previous studies of *Tragopogon* allopolyploids showed that homeolog loss is a common consequence of allopoyploidization, here we show that DNA methylation can silence one progenitor homeolog or it can regulate the level of expression of the two progenitor homeologs. As further genomic resources for *Tragopogon* are developed, genome-wide methylation analyses should be undertaken to assess how extensive homeolog methylation is within the allopolyploid species.

## Methods

### Plant material

DNA for the diploid parents (*Tragopogon dubius* and *T. pratensis*) and *Tragopogon miscellus* used was the same as previous studies [42, 43]. Briefly, DNA was extracted by a modified CTAB method [70] from tissue previously flash-frozen in liquid nitrogen. Leaf tissue was collected from seedlings grown under standardized glasshouse conditions. In total, 17 *T. miscellus* individuals, each of which previously showed gene silencing (TDF-44 in [43]; S2, S3 and S18 in [42]; Table 1), were examined. Three representatives of each diploid species were also included.

### Bisulfite conversion

Prior to bisulfite conversion, genomic DNA of the diploid and polyploid samples was digested with *Eco*RV (New England Biolabs, UK), which does not cut within the genes of interest. Two micrograms of genomic DNA were digested in a total volume of 100 μl with 80 units of *Eco*RV, 10X buffer and 10 μg BSA. The reaction was incubated at 37°C overnight (16-18 hours) and the digested DNA cleaned by ethanol precipitation. Bisulfite conversion was carried out using the EZ DNA Methylation kit (Zymo Research, USA). After bisulfite conversion, the single-stranded DNA was quantified using parameters for RNA-40 on a Nanodrop-1000 (Thermo Fisher Scientific, USA).

### Amplification and sequencing of genomic and bisulfite-Converted DNA

Primers were designed following a home-made genome walking kit [71]. Separate primers were designed to amplify sense and antisense strands, because after bisulfite conversion the two strands were not precisely complementary, with additional primers designed to perform nested PCR, using Methyl Primer Express software v. 1.0 (Applied Biosystems, USA). Primers 26-29 bp in length were designed to generate an amplicon of ~300 bp and with a C or T near the 3’ end to avoid non-specific binding in the bisulfite-converted DNA. The primers used for amplification of genomic DNA and bisulfite-converted DNA are listed in Additional file 1: Table S1.

Amplification of bisulfite-converted DNA for the primary PCR reaction was conducted in a total volume of 25 μl with 10 ng template DNA, 10 μM of both gene-specific forward and reverse primers, 10X PCR buffer, 10 mM dNTPs and 1 unit of Takara Ex Taq^TM^ polymerase (Takara Biotechnology, Japan). Genomic and bisulfite-converted DNA was amplified using the following PCR program: 95°C for 5 min, 95°C for 1 min, 53°C for 1 min, 72°C for 1 min for the first 5 cycles, then 44 cycles with 95°C for 1 min, 48°C for 1 min, 72°C for 1 min, and a final extension at 72°C for 7 min. Using the nested primers, another PCR was performed using the primary PCR product as template. The resulting nested PCR products were run on a 1.5% agarose gel stained with ethidium bromide and examined using a Gel Doc 2000 system (Bio-Rad, UK). For sequencing, PCR products were treated with Exonuclease I (5 units) and Shrimp alkaline phosphatase (0.5 unit) prior to the cycle sequencing reaction using BigDye Terminator v. 3.1 (Applied Biosystems). The purified products were sequenced with both forward and reverse primers on an ABI DNA Analyzer 3770 at Massey Genome Service (Palmerston North, New Zealand). The resulting sequences were assembled and analyzed in Sequencher v. 4.10.1 (Gene Codes Corporation, USA).

Because both parental homeologs in *T. miscellus* polyploids showed CG methylation of the S2 locus, cloning was undertaken to determine the methylation status of the parental copies. PCR products of BS-converted DNA were cloned from *Tragopogon miscellus* individuals 2671-11 and 2688-3 using the TOPO TA cloning kit (Invitrogen, CA, USA). Twelve positive clones per sample were sequenced with T3 and T7 primers using the above-mentioned protocols for sequencing.

### Genome walking

In order to determine the methylation status of the promoter region, 5’genome walking was performed following the GenomeWalker manual (Clontech Laboratories, USA) [72]. Genomic DNA of *Tragopogon dubius* (a diploid parental species) was digested with three different restriction enzymes: *Eco*RV, *Dra*I and *Sca*I (New England Biolabs, USA) in separate reaction tubes containing 2.5 μg of genomic DNA, 80 units of restriction enzyme and 10X buffer (New England Biolabs) in a total volume of 100 μl. Reactions were incubated at 37°C for 16-18 hours. These reactions were ethanol precipitated in the presence of 20 μg glycogen and 3M sodium acetate. Adapter ligation to the precipitated, digested genomic DNA was performed in a total volume of 8 μl containing 25 μM adapter, 10X ligation buffer, 3 units of T4 DNA ligase (New England Biolabs) and 0.5 μg of purified DNA. Primary PCR was performed in 50-μl total volume using 10 mM dNTPs, 10X PCR buffer (Takara Biotechnology, Japan), 10 μM of adapter primer AP1 (Forward) and gene-specific primer (Reverse) (gene-specific reverse primers for all the genes S2, S3, S8, S18 and TDF-44 are listed in Additional file 1: Table S1) and 1 unit of Takara Ex Taq polymerase (Takara Biotechnology, Japan). The PCR profile for the primary PCR was as follows: first 7 cycles at 94°C for 25 sec, 72°C for 3 min, then remaining 32 cycles at 94°C for 25 sec, 67°C for 3 min, and a final extension at 67°C for 7 min. Primary PCR products for the nested round were diluted 1:50 in ddH_2_O. In the secondary PCR, 10 μM nested adapter primer AP2 (forward) and internal gene-specific primers (reverse) were used (Table S1) and 2 μl of diluted primary PCR product were used as template. The secondary PCR profile was as follows: 94°C for 25 sec, 72°C for 3 min for 5 cycles and 94°C for 25 sec, 67°C for 3 min for next 20 cycles, then final extension at 67°C for 7 min. Secondary PCR products were separated on a 1% agarose gel and products from each library were cloned. At least ten positive clones per gene per individual were sequenced. The resulting sequences for each gene were aligned with previously obtained sequences of that gene in Sequencher. New methylation-specific primers were designed to amplify promoter regions from bisulfite-converted DNA. The amplified promoter regions from bisulfite-converted DNA and genomic DNA of all five genes were sequenced for the *T. miscellus* polyploids and the progenitors *T. dubius* and *T. pratensis*.

## Electronic supplementary material

Additional file 1: Table S1: List of primers used for amplification of bisulfite-converted DNA and 5’ genome walking. (XLSX 18 KB)
